# The Importance of a Diagnosis

**Published:** 1890-05

**Authors:** F. S. Davis

**Affiliations:** Quincy, Mass.


### THE IMPORTANCE OF A DIAGNOSIS.

### Illustrated by Cases.

F. S. Davis, M. D., Quincy, Mass.

(Read at the Meeting of the Organon Society, March 5th.)

I was called Tuesday P. M., February 18th, 1890, to William L. Powell, æt. three years, five months (mulatto), of Milton. Had been sick ten days.

Was at first inclined to lie down; complained of headache; face hot, tongue white, skin dry, thirst.

Was given castor-oil by mother, and diarrhœa set in; stools yellow, with tenesmus. No appetite; some nausea.

This condition continued, with higher fever, and Thursday he seemed to have pain all over; more nausea; complained of this aching pain in arms and hands, and was distressed in breathing. Would cry out with pain if he moved in sleep, and when awake. Would frequently cry out a sharp cry as if in pain, but would not tell its location. An allopathic physician was called in; said he could not tell what it was; left some powders, which he said would bring down the fever.

There was no change. Doctor came again; said he could not tell anything about the cause of the pain; would call again; left no medicine. The father went to a drug store and got a powder, made of Sub. nit. of Bismuth and Opium, and they gave one or two of them. No better; dull and stupid; still crying out, particularly if moved; called for nothing but drink; had frequent stools. They sent word to the doctor not to come again, and telephoned for me.

I found the boy stupid, eyes heavy and dull, lips dry and cracked, corners of mouth sore. Tongue *brown and dry*. Temperature 102°, pulse 116, *skin dry, head hot* and painful; *cries if moved*, yet wants *to be taken up*. Stools have been frequent, are yellow, some flatus, with tenesmus and pain. Diagnosis, typhoid rheumatic fever. Bry.$^{200}$, one powder dry, and Sac.-lac.

February 19th, morning.—No fever, pulse 104. Less thirst.

Is very fretful; cries out often; wants to be held, but cries if attempt is made to move him. Frequent yawning; corners of mouth sore; lips covered with dry scabs. Picks at lips, causing bleeding. Eyes heavy and dull. Is so fretful nothing suits him; will cry out if spoken to; cries out in sleep, or when sitting. Feet have been swollen a little for two or three days. Urine highly colored. Pain is complained of in hips and knee. Mother says he complained of pain in legs, and mostly in left knee at first.

Will cry out if legs are moved, also when turning in bed. Continued with Sac.-lac.

Evening, February 19th.—More comfortable when sleeping; less crying out; less thirst. Has taken a little milk (first food taken). Stool only twice; character same. As the boy was sleeping did not take temperature. Continued Sac.-lac.

February 20th, morning.—Better sleep; less crying out in sleep; no stool. *No fever;* has called for an egg, and has eaten half of one with relish. Tongue cleaner. His mother says one half of his face is hot, the other cold; noticed it last evening, and it continues to-day. Has been very fretful. Nothing is right for him. Wants to be held all the time, or carried about. Cham.$^{200}$, one powder.

February 21st.—Better. Only a few times disturbed through the night by crying out. Bowels regular. More appetite. No fever. Tongue is now white as at first of sickness. Water blisters have appeared between the toes. Sac.-lac.

February 23d.—Up and dressed, sitting up; makes no disturbance. Tongue clean. Eyes natural; good appetite. Discharged cured.

The foregoing case of Master P., which I have read, has brought to my mind the question of the necessity of making a correct diagnosis, for answer and discussion.

I believe a diagnosis is important, but far from being necessary for a cure. Had Homœopathy not been able to meet the case on the basis of *totality of symptoms,* I should have been placed at a disadvantage in being unable to decide just the nature of the case, as was the allopathic physician. I would not uphold any negligence of a thorough examination of each case, so that all conditions may be known so far as is possible. I believe it is our duty to take notes of all symptoms carefully, and I think it will quite certainly be found that in this way we shall arrive at a correct understanding of the case. Particularly will this be true if we give to each symptom its true interpretation.

Early in my practice I was called to a Mrs. R., æt. thirty-seven, who had suffered for years from dysmenorrhœa. I was particularly anxious to make a cure with remedies, for the lady was a strong homœopath, and so situated as to be of help to me by her recommendation. I felt quite sure of success, for I got plenty of symptoms, of which I have not now a record. Each time I prescribed I felt I must get a good result, but my failure was as marked as my hope of success had been strong. I confessed to my patient that I could give no reason for my failure, and would only make one more request, and if granted, and it gave no reason for failure I would admit that Homœopathy had failed at my hands. I was permitted to make a vaginal examination, which solved the question.

The neck of the womb was quite firmly united to the vaginal wall on the right side, the cord of attachment was a little less than one-half of an inch in length, and quite firm, and about a fourth of an inch in width. This adhesion accounted for the pain she had suffered "as of something tearing and pulling her to pieces." The neck of the womb could not enlarge or move except she felt inconvenience.

The symptoms were all removed by cutting this attachment.

Our failures to cure after careful study for the right remedy should lead us to still more careful examination of our case before deciding that our system of medicine is at fault.

Let no symptom be overlooked, and we shall be very likely to have our diagnosis with a sure guide to the true remedy.

If careful taking of notes in each case is made the rule, we are prepared for learning by our success what are guiding symptoms, and thus make our experience a sure help not only to ourselves but to others, by publication of these results.

